# Design and clinical validation of a software program for automated measurement of mammographic breast density

**DOI:** 10.1186/s12911-020-1062-y

**Published:** 2020-03-02

**Authors:** Adriano L. C. Araújo, Heliana B. Soares, Daniel F. Carvalho, Roberto M. Mendonça, Antonio G. Oliveira

**Affiliations:** 1 Department of Radiology, Hospital Universitário Onofre Lopes, Universidade Federal do Rio Grande do Norte, Av. Nilo Peçanha 620, Petrópolis, Natal, RN 59012-300 Brazil; 2 Instituto de Radiologia de Natal, Av. Afonso Pena 744 - Tirol, Natal, RN 59020-100 Brazil; 3 Department of Biomedical Engineering, Centro de Tecnologia, Universidade Federal do Rio Grande do Norte, Campus Universitário, Av. Senador Salgado Filho 300, Lagoa Nova, Natal, RN 59078-970 Brazil; 4 Department of Pharmacy, Centro de Ciências da Saúde, Universidade Federal do Rio Grande do Norte, Rua General Gustavo Cordeiro de Farias s/n, Petrópolis, Natal, RN 29012-570 Brazil

**Keywords:** Breast density, Automated measurement, Mammography, Cancer, Reliability

## Abstract

**Background:**

Mammographic breast density is an important predictor of breast cancer, but its measurement has limitations related to subjectivity of visual evaluation or to difficult access for automatic volumetric measurement methods. Herein, we describe the design and clinical validation of Aguida, a software program for automated quantification of breast density from flat mammography images.

**Materials and methods:**

The software program was developed in MatLab. After image segmentation separating the background from the breast image, the operator positions a cursor defining a region of interest on the pectoralis major muscle from the mediolateral oblique view. Then, in the craniocaudal view, the threshold for separation of the dense tissue is based on the optical density of the pectoral muscle, and the proportion of dense tissue is calculated by the program. Mammograms obtained from 2 different occasions in 291 women were used for clinical evaluation.

**Results:**

The intraclass correlation coefficient (ICC) between breast density measurements by the software and by a radiologist was 0.96, with a bias of only 0.67 percentage points and a 95% limit of agreement of 13.5 percentage points; the ICC was 0.94 in the interobserver reliability assessment by two radiologists with different experience; and the ICC was 0.98 in the intraobserver reliability assessment. The distribution among the density classes was close to the values obtained with the volumetric software.

**Conclusions:**

Measurement of breast density with the Aguida program from flat mammography images showed high agreement with the visual determination by radiologists, and high inter- and intra-observer reliability.

## Background

Mammographic breast density is usually expressed as the proportion of dense tissue (fibroglandular tissue) relative to the entire breast (fibroglandular tissue plus adipose tissue). Quantifying breast density is of great importance because it is a primary risk factor for breast cancer [1–9], as well as a cause of false negatives in the mammographic diagnosis of breast cancer [10, 11]. Breast cancer represents the second cause of cancer death among women, with a worldwide annual mortality rate of about 458,000 deaths and an incidence of approximately 1.4 million new cases each year [12]. Breast cancer incidence has increased over the years, possibly related to improvements in imaging methods involved in the early diagnosis, and the greater number of women who undergo this examination every year [13].

It has been shown that the risk of breast cancer associated with breast density is more correlated with the proportion of dense tissue in the breast than it is with its total amount [14]. Accordingly, mammographic breast density has been incorporated as a factor in risk models for breast cancer [15], leading to an increase in the accuracy of the Gail model for Breast Cancer Risk [16–19], and is considered fundamental for elaborating individualized screening or prevention standards for this disease [15]. About half of the American states currently have specific legislation on breast density [20].

The commonly used method for measuring breast density has been visual assessment by a radiologist, and its classification as defined in the ACR (American College of Radiology) BI-RADS® [21] lexicon. However, several informatics tools have been developed in order to decrease the measurement subjectivity and intra- and inter-observer variability. The first proposal was Cumulus®, a software which identifies and quantifies dense breast tissue according to an operator’s interactive selection of a pixel density threshold [22], which still had the limitation of operator subjectivity. Newer methods have proposed fully automatic and volumetric measurement, especially the Volpara® (Volpara Solutions) and Quantra® (Hologic, Danbury, Conn) software programs. However, there is currently no unanimity regarding the most appropriate method for measuring breast parenchyma density [23].

In view of the above, in this article we present the Aguida software program, a computer application for objectively measuring breast density with easy operation. Image thresholding is based on the density of the easily identifiable pectoralis major muscle, which provides a reference value for the automatic image segmentation of the dense tissue and subsequent quantification of breast density, therefore minimizing observer subjectivity.

## Methods

A total of 291 randomly selected women from annual screening programs who had bilateral mammography in the two routine views (MLO and CC) were enrolled in this study. Women with some feature preventing satisfactory visualization of the pectoral muscles in the MLO view in both breasts, such as Poland’s syndrome, paralysis, rotator cuff tear, pacemaker in the axillary region, or those with prostheses or bilateral breast implants were excluded, in addition to cases of malposition. The mean age of the 291 women was 51.5 ± 10.1 years (range 33 to 81 years) and the mean body mass index (BMI) was 27.4 ± 4.55 kg/m^2^ (range 11.0 to 43.2 kg/m^2^).

Two radiologist physicians with different experience profiles participated in the study, one with 15 years of experience in mammary radiology (observer 1) and one with 5 years of experience in general radiology (observer 2).

Bilateral digital mammographic images were obtained in DICOM format from each subject in the mediolateral oblique (MLO) and craniocaudal (CC) routine views, with a Hologic Selenia mammogram (Lorad/Hologic, Danbury, CT, USA). The technicians involved received guidance and the necessary training to ensure good positioning in the CC view, aiming to include all fibroglandular tissue in this incidence as the breast density measurement may vary due to differences in positioning [24].

The approach to developing the Aguida software was based on empirical observation that the density of the pectoralis major muscles is visually correlated to the density of the mammary fibroglandular tissue in mammograms. The software was developed in Matlab (Natick, MA, USA), and capitalizes on the correlation between those two densities to define the threshold for the segmentation of the mammary fibroglandular tissue by multiplying the pectoralis major muscle density as measured with a user-operated Region Of Interest (ROI), with a pre-defined constant amount.

A set of data (vector) is created after selecting the region of interest in the MLO view with an ROI positioned over the image of the pectoralis major muscle, storing the value of all the pixels in the selected region, and then the median optical density of the region is computed. The threshold used to segment the fibroglandular tissue from the adipose breast tissue is obtained by multiplication with a constant value. This constant value was estimated from a sample of 231 randomly selected mammograms, in which it was found that the average ratio of muscle to glandular tissue optical density was 0.72 (95% confidence interval 0.712 to 0.728), and that value was adopted as the multiplication constant.

**Fig. 1:**
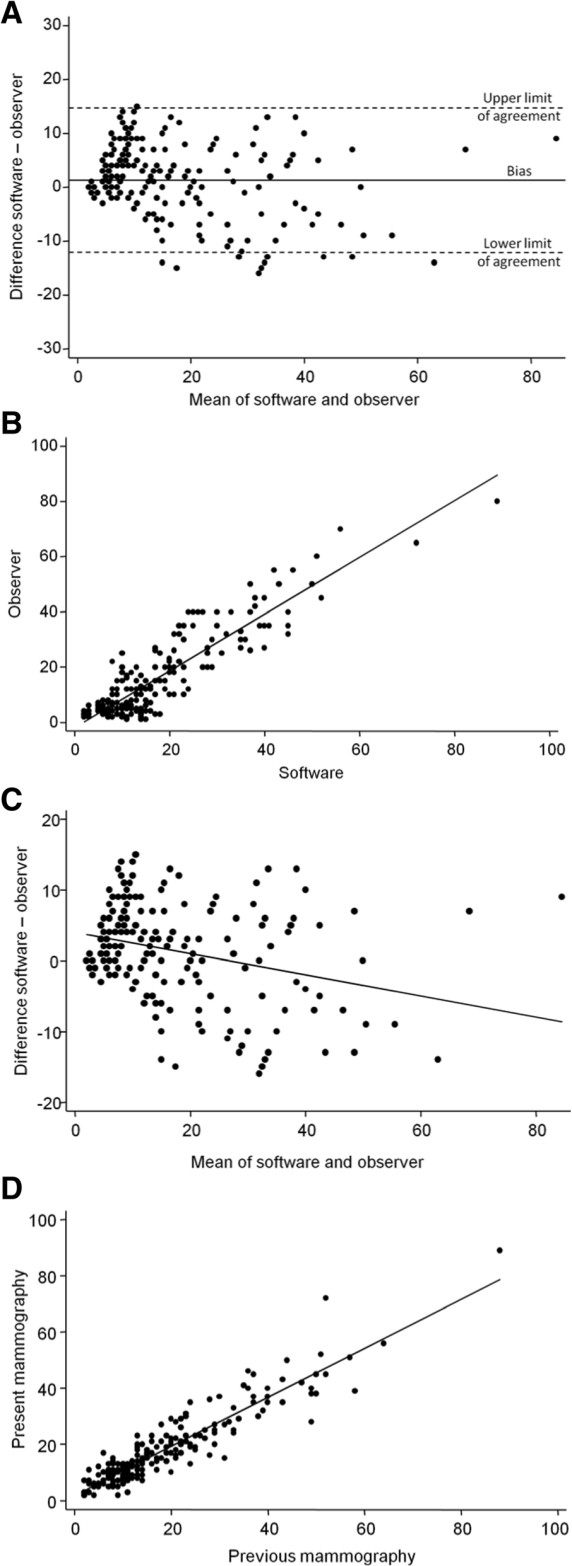
**a** Clinical validation of the Aguida software for automated measurement of mammographic breast density – Agreement between the software and visual assessment; **b** Clinical validation of the Aguida software for automated measurement of mammographic breast density – Correlation between the software and visual assessment; **c**. Clinical validation of the Aguida software for automated measurement of mammographic breast density – Correlation between the difference and the average of visual and software measurements of breast density; **d**. Clinical validation of the Aguida software for automated measurement of mammographic breast density – Reliability of breast density measurements with the software between two mammograms in the same subjects separated by 1 to 3 years

Next, in the CC view, the software separates the breast area from the background, which was defined as all pixels having zero grey-level value (black) which are exclusively surrounded by pixels with zero value. Next, the program identifies the breast area considered dense, meaning the one which contains all the pixels with a value equal to or greater than the threshold value. The pixels corresponding to the points of dense parenchyma are highlighted, creating a mask overlaying the original image and generating a visual return to the radiologist (Fig. 1). Then, still in the CC view, the software computes and displays the dense tissue percentage in relation to the total breast area. The reason for the dense fibroglandular tissue measurement not being performed in the MLO view is because the images of the muscles in this view would also be selected, thus generating a wrong proportion. On the other hand, the pectoral muscles are not commonly shown in the CC view, and when they do show, they appear as a thin band in the posterior region of the breast, so that its proportion in relation to the total breast area is not significant.

Next, the breast density proportions were measured in a sample of 291 new subjects by visual evaluation and by using the software (with a constant of 0.72) blinded to the results of the visual evaluation in order to assess the validity of the software compared to the visual evaluation. Thus, the breast density measurements of those patients were compared to measurements made on a prior exam of the same patients performed no less than 1 year before, being obtained from a digital image file in order to analyze the reliability of the software. The time lag between the two mammograms was limited to 3 years to avoid large changes in the breast structure due to the usual gradual and proportional increase of its adipose component [10, 25]. Women who had undergone a surgical procedure, hormone replacement, or who developed a new finding in both breasts, as well as those who had a weight variation greater than 20% between the two mammograms were excluded from this evaluation, as such factors could generate large differences between the densities being compared [26]. Therefore, reliability was assessed in 282 patients after exclusions.

The evaluation of breast density with the software was performed by observer 1, who was blinded to the subject identification. Then, the breast densities of the 291 mammograms were measured with the software by observer 2 for the inter-observer variability evaluation, who was blinded to the results of the measurements made by observer 1. The radiologists were trained to measure the density of the pectoralis major muscle in its more homogeneous portion, free of accessory glandular tissue, vessels, lymph nodes, skin folds or other additional findings. They were also oriented to visually quantify the proportion of breast tissue by taking into consideration the full mammary area, including the entire area of adipose tissue contained in the subcutaneous tissue and in the retromammary space.

The data are presented as mean ± standard deviation or as absolute and relative frequencies for Statistical analysis. The Bland and Altman method [27] was used in order to assess the validity of the Aguida software compared to visual assessment of breast density. This method is applied in situations where there is no gold standard against which a new method can be compared. It assumes that the average of two measurement methods (visual assessment and Aguida software) will be the best estimate of the true value of what is being measured, and the analysis consists of the correlation of the differences between the values obtained by the two methods with their respective means. If the two methods are measuring the same quantity, it is expected that the differences between their values will only arise by random error, and therefore the differences should have normal distribution, zero mean and should not be correlated with the average of the two measurements. The method also assesses the existence of bias in the measurements of one method over the other, as well as its value, in addition to estimating the limits of agreement, defined as the interval which contains 95% of the differences between the two measurement methods.

The Shapiro-Wilk test was used for testing the normal distribution, and the one sample *t*-test for testing the zero mean of the differences in breast density between visual evaluation and the software. The Pearson’s correlation coefficient was used to evaluate the existence of a correlation between differences in the two methods and their means. The intraclass correlation coefficient (ICC) was calculated in order to obtain a measure of the agreement between the two methods, as well as to evaluate the reliability of the software applied to each of the images at two different moments in time. The kappa concordance coefficient was used to evaluate the adequacy of the software to assign the classification of breast density according to the BI-RADS® system (4th edition) for each image.

A *p*-value less than 0.05 was considered as evidence of statistical significance. All analyzes were performed with Stata 15 software (Stata Corp., Collegue Station, TX, USA).

This study was approved by the institution’s Research Ethics Committee and informed consent in writing was obtained from all participants.

## Results

The mean breast densities were 31.6% ± 26.0 (limits 1 to 90%) for the visual evaluation and 32.3% ± 24.4 (limits 2 to 91%) with the software.

Figure 1a shows the agreement between visual and software evaluations by the Bland and Altman method. The differences between the values obtained by the two methods do not have normal distribution (Shapiro-Wilk test: *z* = 3.22, *p* = 0.0006). Their mean was not statistically different from zero (one sample *t*-test: *t* = 1.65, *p* = 0.10) with an estimated value of the bias of 0.67 percentage points (95% confidence interval (CI) –0.13 to 1.47 percentage points). This means that the software shows a trend to return only slightly higher values than the visual evaluation. The value of the 95% limits of agreement means that the difference between the two methods in breast density will not be greater than 13.5 percentage points in 95% of cases. The intraclass correlation coefficient between visual assessment and the software was 0.96 (95% CI 0.95 to 0.97) (Fig. 1b).

There was a small negative correlation between the difference and the mean visual and software breast density measurements (Pearson correlation coefficient: *r* = − 0.23, *p* = 0.0001) (Fig. 1c), indicating that the observer tends to assign slightly higher values than the software as the breast density increases.

For the software reliability analysis, a comparison was made between the measurements performed by the software for the same subject at two moments in time separated by no more than 3 years and no less than 1 year. There was a strong correlation between the two measurements with an intraclass correlation coefficient of 0.98 (95% CI 0.97 to 0.98) (Fig. 1d).

**Fig. 2:**
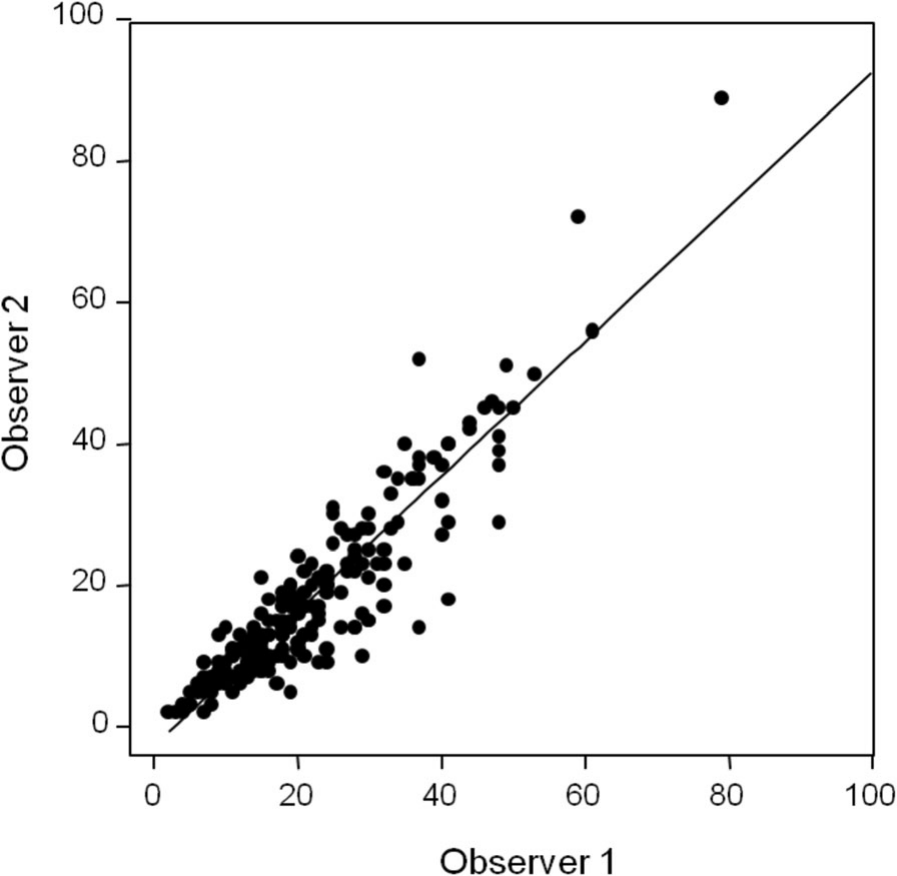
Correlation between breast density measurements obtained by two observers using the software

The agreement for the intraclass correlation coefficient in the comparison between the results obtained with the software operated by two radiologists was 0.94 (95% CI 0.93 to 0.95) (Fig. 2).

The image distribution by the four visual classification types of the BI-RADS® mammary density (4th edition) [28] in types A, B, C and D was 151 (51.9%), 45 (15.5%), 76 (26.1%) and 19 (6.53%), respectively (Table 1). The values found using the Aguida software program were 157 (54.0%), 43 (14.8%), 72 (24.7%) and 19 (6.53%), respectively, with a kappa concordance coefficient of 0.81 (*z* = 20.97, *p* < 0.001). In dividing the findings into dense breasts (breast density > 50%) and non-dense breasts (breast density ≤ 50%), it was observed that 196 (67.4%) were non-dense and 95 (32.6%) were dense by visual evaluation, while 200 (68.7%) were non-dense and 91 (31.3%) were dense with the software. The kappa coefficient was 0.95 (*z* = 16.26, *p* < 0.0001).

**Table 1:** Agreement between BI-RADS classification assessed by visual evaluation and by the Aguida software

		Software				Total
		A	B	C	D	
BI-RADS	A	143	8	0	0	151
	B	14	30	1	0	45
	C	0	5	68	3	76
	D	0	0	3	16	19
Total		157	43	72	19	291

## Discussion

Aguida software is a practical breast density measurement tool, as the only interaction required with the operator is to place a ROI over a suitable portion of the pectoralis major muscle for measuring its optical density. This is a simple and familiar task for any radiologist. The breast image is automatically segmented from the background, thus the running time is short. The breast density is also presented visually in flat images, which is intuitive and familiar to radiologists. Visual density is provided by the software in integers between 1 and 100, and not in four large groups (quartiles) as in conventional visual quantification, which is another advantage because values near the boundary between two categories in a classification into four large density categories may be falsely considered as concordant or discordant [29]. A large portion of subjectivity in measuring breast density is removed by taking the density of the pectoralis major muscle as reference for the entire process.

In the absence of a gold-standard method for measuring breast density, a visual quantification based on the 4th edition of the BI-RADS® was used as reference method because it considers the proportion of dense tissue in relation to the whole breast, in addition to having strong evidence of an association with breast cancer risk [30, 31]. The 5th edition of BI-RADS® no longer quantitatively considers density and therefore brings greater interpretive subjectivity. Our software proposal aims at correcting the subjectivity of the visual classification that is responsible for the moderate inter- and intra-observer agreement indexes seen with that method [21, 31, 32]. Additionally, the comparison between radiologists with different levels of experience in mammography in this study shows that the use of the Aguida software can correct the low agreement in BI-RADS® classification related to the different levels of experience among the evaluators.

The study population had a compatible average age and BMI with that observed in other studies involving patients belonging to mammography screening groups. Regarding the distribution between BI-RADS® density categories A, B, C and D, we obtained breast density values with a significantly lower mean and a different distribution than has usually been reported in other studies [21, 33]. However, despite the apparent disagreement, our values are close to the values of VDB (Volumetric Breast Density) obtained by volumetric software [34, 35]. In those systems, VBD values usually have to be converted to VDGs (Volpara Density Grades) so that they can be correlated with BI-RADS® values [36]. The discrepancy between our findings and those of other studies which used the BI-RADS® classification is possibly explained by the methodology used by the observers in this study for measuring breast density; in much the same way as the software itself, they considered the entire thickness of the subcutaneous tissue and the retromammary space. This is likely to be different from studies which have retrospectively taken values of breast density assigned without this concern and where only glandular tissue had been considered, which might have resulted in overestimating the breast density.

The correlation between software and visual measurements is very high in our study, as can be evaluated by the intraclass correlation coefficient. This degree of agreement is similar to that found in the work of Ciatto et al. [37], who compared density values obtained by Quantra® software (Hologic, Marlborough, MA, USA) and by visual classification, obtaining a concordant classification in 89% of the cases.

The high correlation of the two measurements performed on mammographies taken on two separate occasions in the same subject indicate excellent intraobserver reliability. In addition, it shows that factors which often change between two consecutive examinations, such as degree of exposure, positioning and degree of compression of the mammary tissue [38], do not have a significant impact on breast density measurement by this software.

A very small bias was observed, with the software tending to give a slightly higher value than the visual assessment. The value of the 95% agreement limit was within 13.5 percentage points relative to the visual evaluation. This margin is low when compared to the BI-RADS classification, which is separated by bands of 25 percentage points. There was also a correlation indicating that the observer tends to assign higher values than the software as the density increases, but this correlation is quite small.

A high index of agreement was observed when breast density measurements with the Aguida software were compared between the two observers with different experience, thus suggesting that the software has the ability to reduce the inter-observer variability by adopting the optical density of the pectoral muscle as reference.

Validation of the Aguida software would be more robust with a larger number of radiologists and mammograms, as well as through comparison with volumetric and automatic measurement programs or with Magnetic Resonance. Limitations for its use occur in patients with bilateral breast implants and in pathologies where the pectoralis major muscle is not included in the image, as in cases of severe limitations of movement in the shoulder joint or in the agenesis of the pectoral muscles. However, the bilateral occurrence of those conditions is extremely rare. There may be air pockets forming in the armpit region in the MLO incidence in patients with very low BMI, generating a false decrease in pectoral muscle density. This limitation can be minimized with specific training of the technical team.

Efforts are being made to create secondary constants to obtain density degrees within the tissue considered as dense. This feature may approximate the area-based measurements to those based on the volumetric measurement, since the higher the optical density of a pixel in the flat image, the greater the volume of dense tissue component in that pixel.

## Conclusions

Given the great importance of breast density and the lack of a density measurement method with definitive proof of validity, our findings show that Aguida is a promising tool, since it presented high intra- and inter-observer agreement and excellent reliability, in addition to showing similar results to those obtained with volumetric measurement tools with respect to density values. The use of this tool can present excellent benefits for managing patients, providing an objective parameter to define additional screenings or even for breast cancer prevention, reducing the mortality of this disease and fostering better utilization of resources and efforts. In addition, the software is easy to use and provides simple and fast results, and additionally provides visual feedback to the radiologist. This software can also be used in the educational field, serving as an excellent tool to train radiologists, since it eliminates the need for experience linked to subjectivity. Further studies are needed to substantiate these findings.

## Data Availability

The datasets used and/or analyzed during the current study are available from the corresponding author upon reasonable request.

## Abbreviations

ACR: American College of Radiology
BIRADS: Breast Imaging-Reporting and Data System
BMI: Body mass index
CC: Craniocaudal
DICOM: Digital Imaging and Communications in Medicine
ICC: Intraclass correlation coefficient
MLO: Mediolateral oblique
ROI: Region of interest
VBD: Volumetric Breast Density
VDGs: Volpara Density Grades

## References

[1] Wolfe JN (1976). Risk factors for breast Cancer development determined by mammographic parenchymal pattern. Cancer.

[2] Lee CI, Chen LE, Elmore JG (2017). Risk-based breast Cancer screening: implications of breast density. Med Clin North Am.

[3] Boyd NF, Byng JW, Jong RA, Fishell EK, Little LE, Miller AB, Lockwood GA, Tritchler DL, Yaffe MJ (1995). Quantitative classification of mammographic densities and breast cancer risk: results from the Canadian National Breast Screening Study. J Natl Cancer Inst.

[4] Boyd NF, Lockwood GA, Martin LJ, Knight JA, Byng JW, Yaffe MJ, Tritchler DL (1998). Mammographic densities and breast cancer risk. Breast Dis.

[5] Kerlikowske K, Ichikawa L, Miglioretti DL, Buist DSM, Vacek PM, Smith-Bindman R, Yankaskas B, Carney PA, Ballard-Barbash R (2007). Longitudinal measurement of clinical mammographic breast density to improve estimation of breast cancer risk. J Natl Cancer Inst.

[6] Huo CW, Chew GL, Britt KL, Ingman WV, Henderson MA, Hopper JL, Thompson EW (2014). Mammographic density - a review on the current understanding of its association with breast cancer. Breast Cancer Res Treat.

[7] Vachon CM, van Gils CH, Sellers TA, Ghosh K, Pruthi S, Brandt KR, Pankratz VS (2007). Mammographic density, breast cancer risk and risk prediction. Breast Cancer Res.

[8] Sherratt MJ, McConnell JC, Streuli CH (2016). Raised mammographic density: causative mechanisms and biological consequences. Breast Cancer Res.

[9] Sickles EA (2010). The use of breast imaging to screen women at high risk for cancer. Radiol Clin N Am.

[10] Byng JW, Yaffe MJ, Jong RA, Shumak RS, Lockwood GA, Tritchler DL, Boyd NF (1998). Analysis of mammographic density and breast Cancer risk from digitized. Radiographics.

[11] Kerlikowske K, Zhu W, Tosteson ANA, Sprague BL, Tice JA, Lehman CD, Miglioretti DL (2015). Identifying women with dense breasts at high risk for interval cancer a cohort study. Ann Intern Med.

[12] Ferlay J, Shin HR, Bray F, Forman D, Mathers C, Parkin DM (2010). Estimates of worldwide burden of cancer in 2008: GLOBOCAN 2008. Int J Cancer.

[13] Ban KA, Godellas CV (2014). Epidemiology of breast Cancer. Surg Oncol Clin N Am.

[14] Pettersson A, Graff RE, Ursin G, Dos Santos SI, McCormack V, Baglietto L, et al. Mammographic density phenotypes and risk of breast cancer: A meta-analysis. J Natl Cancer Inst. 2014;106(5). 10.1093/jnci/dju078. 10.1093/jnci/dju078 PMC4568991 24816206

[15] Howell A, Anderson AS, Clarke RB, Duffy SW, Evans DG, Garcia-Closas M, Gescher AJ, Key TJ, Saxton JM, Harvie MN (2014). Risk determination and prevention of breast cancer. Breast Cancer Res.

[16] Tice JA, Cummings SR, Ziv E, Kerlikowske K (2005). Mammographic breast density and the Gail model for breast cancer risk prediction in a screening population. Breast Cancer Res Treat.

[17] Tice JA, Cummings SR, Smith-Bindman R, Ichikawa L, Barlow WE, Kerlikowske K (2008). Using clinical factors and mammographic breast density to estimate breast cancer risk: development and validation of a new predictive model. Ann Intern Med.

[18] Barlow WE, White E, Ballard-Barbash R, Vacek PM, Titus-Ernstoff L, Carney PA, Tice JA, Buist DS, Geller BM, Rosenberg R, Yankaskas BC, Kerlikowske K (2006). Prospective breast cancer risk prediction model for women undergoing screening mammography. J Natl Cancer Inst.

[19] Chen J, Pee D, Ayyagari R, Graubard B, Schairer C, Byrne C, Benichou J, Gail MH (2006). Projecting absolute invasive breast cancer risk in white women with a model that includes mammographic density. J Natl Cancer Inst.

[20] Sprague BL, Conant EF, Onega T, Garcia MP, Beaber EF, Herschorn SD, Lehman CD, Tosteson AN, Lacson R, Schnall MD, Kontos D, Haas JS, Weaver DL, Barlow WE (2016). Variation in mammographic breast density assessments among radiologists in clinical practice: a multicenter observational study. Ann Intern Med.

[21] D’Orsi C, Sickles E, Mendelson E, Morris E (2013). ACR BI-RADS atlas, breast imaging reporting and data system.

[22] Byng JW, Boyd NF, Fishell E, Jong RA, Yaffe MJ (1994). The quantitative analysis of mammographic densities. Phys Med Biol.

[23] Brandt KR, Scott CG, Ma L, Mahmoudzadeh AP, Jensen MR, Whaley DH, Wu FF, Malkov S, Hruska CB, Norman AD, Heine J, Shepherd J, Pankratz VS, Kerlikowske K, Vachon CM (2016). Comparison of clinical and automated breast density measurements: implications for risk prediction and supplemental screening. Radiology.

[24] Kopans DB (2008). Basic physics and doubts about relationship between Mammographically determined tissue density and breast Cancer risk. Radiology.

[25] Lokate M, Stellato RK, Veldhuis WB, Peeters PHM, Van Gils CH (2013). Age-related changes in mammographic density and breast cancer risk. Am J Epidemiol.

[26] Vachon CM, Kushi LH, Cerhan JR, Kuni CC, Sellers TA (2000). Association of Diet and Mammographic Breast Density in the Minnesota breast Cancer family cohort Association of Diet and Mammographic Breast Density in the Minnesota breast Cancer family cohort. Cancer Epidemiol Biomark Prev.

[27] Martin Bland J, Altman D (1986). Statistical methods for assessing agreement between two methods of clinical measurement. Lancet.

[28] D’Orsi C, Sickles E, Mendelson E (2003). Breast imaging reporting and data system. In: American College of Radiology. Breast imaging reporting and data system (BI-RADS® ).

[29] Ko SY, Kim E-K, Kim MJ, Moon HJ (2014). Mammographic density estimation with automated volumetric breast density measurement. Korean J Radiol.

[30] Jeffers AM, Sieh W, Lipson JA, Rothstein JH, McGuire V, Whittemore AS, Rubin DL (2017). Breast Cancer risk and mammographic density assessed with Semiautomated and fully automated methods and BI-RADS. Radiology.

[31] Boyd N, Martin L, Gunasekara A, Melnichouk O, Maudsley G, Peressotti C, Yaffe M, Minkin S (2009). Mammographic density and breast cancer risk: evaluation of a novel method of measuring breast tissue volumes. Cancer Epidemiol Biomark Prev.

[32] Gard CC, Aiello Bowles EJ, Miglioretti DL, Taplin SH, Rutter CM (2015). Misclassification of breast imaging reporting and data system (BI-RADS) mammographic density and implications for breast density reporting legislation. Breast J.

[33] Lee HN, Sohn YM, Han KH (2015). Comparison of mammographic density estimation by Volpara software with radiologists’ visual assessment: analysis of clinical-radiologic factors affecting discrepancy between them. Acta Radiol.

[34] Gweon HM, Youk JH, Kim JA, Son EJ (2013). Radiologist assessment of breast density by BI-RADS categories versus fully automated volumetric assessment. AJR Am J Roentgenol.

[35] Gubern-Mérida A, Kallenberg M, Platel B, Mann RM, Martí R, Karssemeijer N (2014). Volumetric breast density estimation from full-field digital mammograms: a validation study. PLoS One.

[36] Highnam R, Sauber N, Destounis S. Breast density into clinical practice. Breast Imaging. 2012:466–73. 10.1007/978-3-642-31271-7_60.

[37] Ciatto S, Bernardi D, Calabrese M, Durando M, Gentilini MA, Mariscotti G, Monetti F, Moriconi E, Pesce B, Roselli A, Stevanin C, Tapparelli M, Houssami N (2012). A first evaluation of breast radiological density assessment by QUANTRA software as compared to visual classification. Breast.

[38] Alonzo-Proulx O, Mawdsley GE, Patrie JT, Yaffe MJ, Harvey JA (2015). Reliability of automated breast density measurements. Radiology.

